# A Brief History of Cerebellar Neurostimulation

**DOI:** 10.1007/s12311-021-01310-2

**Published:** 2021-08-17

**Authors:** Gustavo V. Ponce, Jana Klaus, Dennis J. L. G. Schutter

**Affiliations:** Department of Experimental Psychology, Helmholtz Institute, Utrecht University, Heidelberglaan 1, 3584CS Utrecht, The Netherlands

**Keywords:** Cerebellum, Deep brain stimulation, History, Research, Transcranial direct current stimulation, Transcranial magnetic stimulation

## Abstract

The first attempts at using electric stimulation to study human brain functions followed the experiments of Luigi Galvani and Giovanni Aldini on animal electricity during the eighteenth century. Since then, the cerebellum has been among the areas that have been studied by invasive and non-invasive forms of electrical and magnetic stimulation. During the nineteenth century, animal experiments were conducted to map the motor-related regions of cerebellar cortex by means of direct electric stimulation. As electric stimulation research on the cerebellum moved into the twentieth century, systematic research of electric cerebellar stimulation led to a better understanding of its effects and mechanism of action. In addition, the clinical potential of cerebellar stimulation in the treatment of motor diseases started to be explored. With the introduction of transcranial electric and magnetic stimulation, cerebellar research moved to non-invasive techniques. During the twenty-first century, following on groundbreaking research that linked the cerebellum to non-motor functions, non-invasive techniques have facilitated research into different aspects of cerebellar functioning. The present review provides a brief historical account of cerebellar neurostimulation and discusses current challenges and future direction in this field of research.

## Introduction

Recent decades has shown a rapid increase of the scientific literature that examines the functions of the cerebellum with neurostimulation. The growth of this field reflects an emerging interest in the cerebellum as a structure that is involved in motor and non-motor functions. The cerebellum is a brain structure located in the posterior fossa of vertebrates. While best known for its role in fine motor processes and balance, increasing evidence points towards the involvement of the cerebellum in cognitive and affective processes as well [1–4]. The introduction of non-invasive brain stimulation (NIBS) techniques like transcranial magnetic stimulation (TMS) and transcranial direct current stimulation (tDCS) provided new ways of investigating the human cerebellum in a non-invasive manner. The ability of these techniques to safely modulate cerebellar neural activity combined with modern neuroimaging techniques and behavioral paradigms has allowed researchers to systematically study the functional neuroanatomy of the cerebellum in healthy volunteers and clinical populations. In recent years, cerebellar neurostimulation has become a routinely used approach to address cognitive and affective neuroscientific research questions and is explored as a possible therapeutic intervention for the treatment of motor-related disorders and psychopathology [5, 6]. The aim of this review is to provide a brief history of cerebellar neurostimulation and how this approach contributed to our knowledge on the functions of the cerebellum.

## 1700–1850: Electric Stimulation as a New Method to Study Human Cerebellar Functions

Before the eighteenth century, knowledge of cerebellar functions was largely based on theoretical speculations and gross anatomical observations [7]. The first experimental attempt at elucidating the functions of the cerebellum was likely performed by Du Verney in 1673, who kept pigeons alive for a short period after ablating their cerebellum to document their behavior. Experimentally ablating or lesioning the cerebellum of animals quickly became the preferred method of cerebellar research. A new method to study cerebellar functions arose from the works of Luigi Galvani (1737–1798). Galvani’s observation that electric sparks induced muscle contractions in frogs’ legs led him to propose that the brain generated electricity, and that this electricity was distributed via the nerves to the muscles by triggering so-called natural electricity [8]. The first publications of Galvani’s observations and experiments on natural electricity were received with enthusiasm. Consequently, Galvani’s work fostered the use and application of electricity in research. Giovanni Aldini (1762–1834), Galvani’s nephew and collaborator, continued to work on natural electricity. In 1798, he initiated a series of experiments on warm-blooded animals. In one of his studies, he demonstrated motor responses following electric stimulation of the cerebellum and corpus callosum of an ox [9].

The early comparative anatomical and physiological approach to the functions of the cerebellum enabled a more satisfactory analysis of cerebellar disturbances in humans [10]. Vincenzo Malacarne (1744–1816), an Italian professor of medicine, surgery, and obstetrics, was the first to provide a complete description of the human cerebellum. Interestingly, Malacarne proposed an alternative to ablation practice in which he examined morphology, such as the number of folia to study cerebellar functions [11]. In addition, he was one of the first to propose environmental effects on cerebellar development. By the turn of the nineteenth century, the basic neuroanatomy of the cerebellum regarding descriptions of the classic nomenclature for the lobules of the human cerebellum and the dentate nucleus had been well documented [12]. By contrast, the functions of the cerebellum were still largely unknown. Luigi Rolando (1773–1831) was among the first to pioneer the use of electricity to study cerebellar functions. Rolando observed that galvanic currents applied to the cerebellum of animals elicited convulsions. He also observed that when the cerebellum of a goat was removed, the animal could no longer stand up or move [12, 13]. These observations led him to the idea that the cerebellum exerts control over motor functions through nerve fluid from the cerebellum. He believed that by using this electric fluid, the cerebellum performed the function of an electromotor or an electric battery [14]. While Rolando overestimated the role of the cerebellum as the only source for motor control of motor functions, the importance of his work lies in the demonstration of a direct relationship between the cerebellum and motor function, making him among the founders of modern cerebellar physiology [12].

Due to the technological and methodological limitations, electric stimulation was still an unreliable approach to study brain functions during this period. Many experiments failed to observe motor responses following stimulation of cortical regions of the brain and cerebellum while other studies found that electric stimulation of the brain could generate movement even after ablation of cerebral cortices [15]. Consequently, cortices were considered largely unexcitable [16]. Further, due to the then prevalent belief that the center of motor functions was located in subcortical regions such as the brainstem or basal ganglia, motor responses observed by experiments stimulating cortical areas of the brain and cerebellum were attributed to electric spread into deeper and neighboring structures. This idea limited the information that the neurophysiologists of the period could infer from early electric stimulation studies. Therefore, most cerebellar neurophysiological research was performed using the ablation method.

## 1850–1920: Discovery of the Effects of Electric Cerebellar Stimulation on Motor Processes

The use of electric stimulation in neurophysiological research regained popularity during the second half of the nineteenth century. Groundbreaking research like the discovery of the motor cortex through electric stimulation [17] prompted interest in the use of electric stimulation to study the cerebellum. To avoid previous criticism, scientists of the time began a methodologically driven investigation of the effects of electric stimulation on the cerebellum [18]. Towards the end of the nineteenth century, the inhibitory effect of electric stimulation of the cerebellar cortex on several motor functions was discovered [19]. This important finding was complemented by subsequent research demonstrating the ability of electric cerebellar stimulation to facilitate motor functions [20].

In their now classic experiment, German neurophysiologists Gustav Fritsch (1838–1927) and Eduard Hitzig (1838–1907) removed the cranium of a dog and stimulated different cortical areas by administering brief electric pulses using a battery and platinum wires. Their study demonstrated that stimulation of the anterior parts of the cerebral cortex elicited muscle movements [17]. Fritsch and Hitzig’s discovery of the motor cortex was important for cerebellar research for several reasons. First, their findings demonstrated the electrical excitability of cortical regions. Second, it was the first evidence for a cortical region involved in motor functions. Finally, the data pointed towards a topographically organized representation of the body in the brain [21]. Consensus among scientists that the cerebellum was involved in motor functions prompted physiologists to investigate whether a topographical representation of the body, similar to that found in the motor cortex, was also present in the cerebellum [22].

David Ferrier (1843–1928) was eager to replicate and extend the findings of Fritsch and Hitzig by attempting to map the cerebellum and other cortical regions with electric stimulation. He began to systematically study brain functions using different excitation methods, including mechanical, faradic, and galvanic current stimulation. In his book *The functions of the brain* [18], Ferrier reported on the overall minimal effect of cerebellar stimulation, often in the form of eye movements and muscle twitches, in fish, monkeys, cats, dogs, rabbits, and pigeons [18, 23]. Some of Ferrier’s observations on the cerebellum were later attributed to current spread to distal regions including the brain stem, rather than as a direct response to cerebellar stimulation. For instance, Ten Cate [24] reported that in fish, bipolar faradization of the cerebellum would only lead to observable movements when the intensity of the stimulation was high. This was a common criticism on the work by Ferrier, Fritsch, Hitzig, and other researchers, with contemporary researchers attributing the reported findings to artifactual current spread into deeper brain structures [15, 25]. This meant that the observed effects of cerebellar stimulation could potentially be cofounded by additional stimulation of subcortical regions.

To address these concerns, neurophysiologist Victor Alexander Hadden Horsley (1857–1916) stimulated the cerebral and cerebellar cortex using the minimum amount of stimulation necessary to attain observable movement [21]. This resulted in a considerable reduction of previously used stimulation intensities and avoided potential stimulation of deeper structures. In 1895, Horsley’s colleague Max Solly Löwenthal (1867–1960) observed that when both cerebral hemispheres of dogs and cats were removed, inhibition of the elicited decerebrate muscle rigidity could be attained by faradization of the anterior surface of the cerebellum. He noticed that muscle relaxation only persisted as long as the stimulation was administered [26]. Löwenthal and Horsley replicated this observation over many experiments, eventually showing that the observed effects of stimulation could be localized to defined areas of the cerebellar cortex. Specifically, stronger inhibitory motor effects were observed in the ipsilateral limbs of the stimulated cerebellar hemisphere, while the most excitable area was at the junction of the vermis superior of the lateral lobe. The findings added to the initial finding of the inhibitory effect of electric cerebellar stimulation. Notably, Charles Scott Sherrington (1857–1952) had independently made the same discovery and reported his observation to the Royal Society a week later than Löwenthal and Horsley [19].

Sherrington moved on to other research topics while only making small mention of his findings in later studies [19, 27]. Horsley, however, continued cerebellar research and was involved in developing a rudimentary stereotactic frame for use in animals, which helped to demonstrate electrical excitability of deep brain structures by using surface landmarks [23].

Löwenthal, Horsley, and Sherrington’s discoveries were relevant for several reasons. Their finding demonstrated the inhibitory effect of cerebellar stimulation and confirmed the electric excitability of the cerebellum. Furthermore, their work provided evidence for a functional organization of the cerebellar cortex, a concept contrary to the then prevalent belief that the cerebellum worked as a single unit. Yet, the importance of these findings went largely unnoticed at the time.

The first account of the *facilitatory* effect of cerebellar stimulation was documented by Rossi [20]. Rossi noticed that the threshold to effectively stimulate the motor cortex was consistently lower when it was combined with contralateral faradic stimulation of the cerebellar cortex. However, similar to Löwenthal, Horsley, and Sherrington’s discovery, replication and validation of this finding would not occur until many years later.

## 1920–1960: Research of Cerebellar Stimulation Properties

During the twentieth century, validation of previous findings through replication studies [28, 29] led to more systematic research of the effects of cerebellar stimulation and the parameters that could influence the outcome of stimulation [30]. In the second half of the twentieth century, the frequency-dependent effect of cerebellar stimulation was established in the literature [31], while emerging evidence from animal studies [32] and stereotactic neurosurgery promoted the concept of using invasive electric stimulation to treat motor and psychiatric disorders.

### Electric Cerebellar Stimulation

General acceptance by the scientific community of the inhibitory effects of cerebellar stimulation did not occur until Miller and Banting [29], along with Bremer [28], reproduced the preliminary accounts described by Löwenthal, Horsley, and Sherrington over two decades prior. By using low-intensity bipolar stimulation and thus preventing potential current to spread into deeper regions of the cerebellum, these two studies demonstrated that the inhibitory response from cerebellar stimulation indeed originated from the cerebellar cortex. Because electrical stimulation of the dorsal spinal columns could also lead to inhibition of decerebrate rigidity [19], the Belgian neurophysiologist Frédéric Bremer (1892–1982) further investigated the nature of the cerebellar inhibitory response. His research showed that while spinal inhibitory responses in decerebrate rigidity could be reversed by the injection of the alkaloid strychnine, no changes were observed for cerebellar inhibitory responses [33]. This dissociation indicated that cerebellar inhibition is a distinct physiological phenomenon. A replication of the work by Rossi [20] provided further support for facilitatory effects of cerebellar stimulation [34]. These studies caused a sharp rise in research devoted to stimulating different parts of the cerebellum.

During investigations of the effects of cerebellar stimulation on movements induced by electrical stimulation of the motor cortex, researchers noticed that in some cases, stimulation led to motor facilitation instead of inhibition [35]. Addressing the inconsistencies surrounding cerebellar stimulation, Nulsen, Black, and Drake [30] conducted an experiment in cats, dogs, and chimpanzees in which they showed that movement elicited by motor cortical stimulation could be either inhibited or facilitated depending on the frequency of subsequent electric stimulation of the anterior cerebellum. They commented that increased frequency would lead to facilitation whereas reduced frequency would lead to inhibition. However, the report neither specified details on the frequency range, nor the precise areas of stimulation, limiting its experimental usefulness [22].

The Italian scientist Giuseppe Moruzzi (1910–1986) conducted a number of experiments with electrical stimulation and provided the first evidence for the involvement of the cerebellum in homeostatic functions and emotions. He showed that the somatic and visceral components of hypothalamus-induced sham rage in cats could be inhibited by electric stimulation of the cerebellar vermis [36]. Similarly, Moruzzi’s work expanded on the preliminary findings of the frequency-dependent response of cerebellar stimulation. By refining prior studies on the subject, he researched the effects of cerebellar stimulation on decerebrated rigidity. Eventually, he demonstrated that the effects of stimulation on the same cerebellar region could be reversed from inhibitory to facilitatory by lowering the frequency of the stimulation [31]. He also provided better defined stimulation frequency ranges for obtaining reliable effects, with stimulation rates of 50 to 300 Hz eliciting inhibition, stimulation rates of 2 to 30 Hz eliciting facilitation, and intermediate responses occurring at frequencies between 30 and 50 Hz. Intermediate responses constituted a weaker facilitatory response occurring around 30 Hz, neither facilitatory nor inhibitory responses occurring at 40 Hz, and a clear inhibitory response occurring at 50 Hz.

Parallel to Moruzzi’s work, other studies reported on the clinical potential of cerebellar stimulation. Cooke and Snider [32] found that stimulation of the cerebellar cortex could alter neural discharges of electrically induced cerebral seizures in cats. Similarly, Ito, Yoshida, and Obata [37] performed intracellular recordings in cats to measure inhibitory postsynaptic potentials elicited by cerebellar stimulation. The results of their study implied that the output of cerebellar Purkinje cells was exclusively inhibitory, thus shedding more light on the underlying mechanisms of the inhibitory effects of cerebellar stimulation. Furthermore, advancements in neurosurgical techniques such as the use of electric stimulation to locate and target brain region before clinical interventions provided accounts of the potential clinical usefulness of deep electric stimulation. For instance, neurosurgeons noticed that high-frequency stimulation delivered to relevant areas like the globus pallidus could diminish motor dysfunctions such as tremor intensity in Parkinson’s disease (PD) [38]. Subsequent research investigating the clinical potential of electrical stimulation provided evidence for the efficacy of deep electrical stimulation in the treatment of dyskinesia and PD [39, 40].

These studies and clinical observations would ultimately serve as the base for the implementation of cerebellar stimulation in the clinical practice.

## 1960–2000: Cerebellar Stimulation in the Clinical Practice and the Introduction of Non-Invasive Stimulation Methods

Following up on the clinical potential of deep electric stimulation, chronic cerebellar stimulation was introduced as a potential treatment for epilepsy and cerebral palsy [41, 42] as well as psychiatric disorders [43]. However, results within this line of research turned out to be difficult to replicate. In spite of the poor replicability, this period of experimentation introduced the potential of cerebellar stimulation not only as a research tool but as a potential clinical therapy. In the last decades of the twentieth century, non-invasive neuromodulation techniques were introduced. Towards the end of the century, a series of studies demonstrated the capacity of transcranial electric stimulation and TMS to modulate cerebellar excitability.

### Invasive Cerebellar Stimulation in the Treatment of Motor Dysfunctions

Cerebellar DBS in the clinical setting was pioneered by the American neurosurgeon Irving S. Cooper (1922–1985), whose career and interest were centered around the treatment of motor diseases. During the early 1970s, Cooper started to place electrodes over the superior and anterior parts of the cerebellar cortex in an attempt to treat disorders such as epilepsy [42] and cerebral palsy with spasticity [41]. Cooper’s rationale was based on Moruzzi’s work on cerebellar stimulation and the discovery of the inhibitory nature of Purkinje cells by Ito, Yoshida, and Obata [37]. The procedure involved implanting electrodes on the surface of the cerebellum and delivering chronic high-frequency stimulation, allowing to successfully manipulate the inhibitory mechanisms of cerebellar stimulation therapeutically [44]. Criticism of the underlying physiological rationale for Cooper’s procedures arose from earlier studies suggesting that Purkinje cells neighboring the electrode arrays were actually inhibited [45, 46]. The safety of the procedure was also questioned by Bensman and Szegho (47) who cited a histological study in monkeys exhibiting decreased numbers of Purkinje cells and levels of the inhibitory neurotransmitter γ-aminobutyric acid (GABA) after cerebellar stimulation [47]. Furthermore, the reliability of Cooper’s procedures was also brought into question as several double-blind studies failed to replicate his initial findings. In cerebral palsy patients, the majority of studies reported weak results following chronic cerebellar stimulation, listing factors like electrode placement, stimulation intensity, and patient selection as potential reasons (49–54). The lack of replicability of Cooper’s findings resulted in a decline of cerebellar stimulation as a treatment for cerebral palsy during the 1980s [48]. Likewise, Cooper’s use of cerebellar stimulation in the treatment of epilepsy suffered from poor replicability in double-blind studies [49, 50]. Nonetheless, Cooper continued to work on cerebellar stimulation and after 25 years of experience concluded that chronic cerebellar stimulation as a therapeutic tool produced modest results. He commented that to obtain meaningful effects, it was important to select appropriate patients and to closely monitor them, alluding to differences in etiology and pathophysiology of diseases like cerebral palsy [51, 52].

Despite the controversy surrounding Cooper’s work, the use of cerebellar stimulation to treat motor dysfunctions associated with cerebral palsy continued to be promoted by some neurosurgeons well into the 2000s. Although not as impressive as Cooper had originally reported, chronic cerebellar stimulation led to improvements in up to 75% of patients treated, with studies reporting reduction in symptoms like spasticity [41, 53–55], gait [56], and respiratory function [57–59]. Additionally, some of these studies reported psychological improvements including reduced anxiety and improved visuomotor functions [60–62].

### Invasive Cerebellar Stimulation to Modulate Behavior

Invasive cerebellar stimulation was briefly implemented in behavioral modulation during its early years. The American neurosurgeon Robert G. Heath (1915–1999) and his colleagues conducted a series of recordings in laboratory animals and some psychiatric patients, which led them to believe that the cerebellum was functionally connected to the limbic system. Consequently, Heath argued that the deep cerebellar nuclei (DCN) including the fastigial nucleus played an important role in affective processing [63–65]. Based on these observations, Heath implanted electrode arrays in the anterior and posterior surface of the cerebellum to treat a series of behavioral disorders including schizophrenia, depression, epilepsy with behavioral pathologies, and patients with severe brain damage. His follow-up publication to these case reports showed behavioral improvements in patients suffering from depression, epilepsy, and brain damage [43, 66].

Although Heath reported promising results, his work on cerebellar stimulation was never followed up. This may have been due to the decline of neurosurgical procedures for psychiatric diseases following the anti-psychiatric movement and the introduction of pharmacological alternatives during the 1950s, as well as the controversial status of some of Heath’s work [67]. Nevertheless, Heath’s work was the first attempt at using cerebellar stimulation to modulate behavior.

### Non-Invasive Stimulation Methods in Cerebellar Research

Observations from stereotactic neurosurgery procedures during the previous decades increased interest in the experimental and clinical potential of invasive stimulation methods. Further, this also led to research focused on finding non-invasive alternatives of stimulation. During the 1960s, animal studies demonstrated that constant direct current stimulation over the skull had the capacity to alter cortical excitability in a polarity-dependent manner. Cathodal stimulation generally led to neural hyperpolarization, thus decreasing cerebral excitability, and anodal stimulation exhibited the opposite effect [68, 69]. Transcranial polarization was soon explored as a possible treatment for patients suffering from treatment-resistant depression and schizophrenia, with beneficial effects observed in the former [70–75]. The technique was gradually abandoned during the 1970s due to the rise of pharmacological alternatives to treat psychiatric diseases [76]. While interest in transcranial polarization declined during this period, a different form of non-invasive stimulation was introduced by Anthony Barker and his colleagues at the University of Sheffield in 1976 [77], namely transcranial magnetic stimulation. The main principle of TMS is based on the works of Michael Faraday on electromagnetism from 1831. Faraday’s work using magnets and electric coils demonstrated that a changing magnetic field creates an electric current flow in conductive material. Barker and his colleagues successfully adapted these principles to neurons and in 1985, they introduced the first reliable TMS machine [77].

During the last decade of the twentieth century, both electric and magnetic methods were used to target the cerebellum non-invasively. Britton et al. [78] and Ugawa et al. [79] made use of a (transcranial) polarization method developed by Merton and Morton [80] to stimulate the human cerebellum through the scalp. This technique used a single high-voltage discharge to overcome the protective electrical resistance of the scalp and skull [81]. To quantify the effect of electric cerebellar neurostimulation, they measured electromyographic (EMG) responses elicited by magnetic stimulation of the motor cortex. Britton and colleagues [78] and Ugawa and colleagues [79] successfully demonstrated that cerebellar transcranial electric stimulation altered motor responses as evidenced by lower EMG responses in the forearm flexor and first dorsal interosseous muscles. Ugawa et al. [79] suggested that electric stimulation activated the cortical output of Purkinje cells leads to inhibition of cerebello-thalamo-cortical projections. Although successful in modulating motor responses, the electric stimulation technique used in these studies elicited painful muscle contractions in the neck. To avoid pain and discomfort of participants, subsequent studies attempted to replicate the phenomena of transcranial cerebellar activation with magnetic stimulation [82]. Cerebellar TMS demonstrated effects comparable to those observed with Merton and Morton’s technique [82–84]. During the second half of the decade, cerebellar TMS was used to establish the involvement of the posterior cerebellum in controlling visually guided saccades [85] and smooth eye pursuit [86]. Furthermore, the clinical potential of cerebellar TMS began to be explored in motor dysfunctions. Shimizu et al. [87], for instance, reported improvement of ataxia gait following cerebellar TMS in patients with hereditary spinocerebellar degeneration and introduced a line of research that would continue during the next century.

## 2000–Present: Cerebellar Neurostimulation in the Twenty-First Century

Cerebellar neurostimulation research during the first two decades of the twenty-first century has been primarily dominated by TMS and more recently tDCS studies. Continuing the groundbreaking research of previous decades in which the cerebellum was implicated in non-motor functions [3], the field of cerebellar stimulation has branched off in different research areas addressing unique aspects of cerebellar functions. Furthermore, clinical research into the therapeutic potential of cerebellar stimulation has also gained considerable interest by researchers and clinicians due to the increasing evidence of cerebellar involvement in several neurological disorders.

### Cerebellar TMS in the Twenty-First Century

During the previous decade, experimental work established the ability of TMS to safely alter cerebellar activity in healthy participants. Building on the experimental work of Ugawa and colleagues [79, 84], TMS protocols were developed to study cerebellar functional connectivity. Similarly, different TMS protocols have been applied to the cerebellum to examine the effects of different stimulation parameters on cerebellar connectivity. Furthermore, cerebellar TMS during the twenty-first century has contributed significantly to the study of cerebellar involvement in motor, cognitive, and affective processes as well as the study of stimulation-induced clinical benefits in diseases such as PD, spinocerebellar ataxia, essential tremors, cervical dystonia, and schizophrenia.

#### Functional Connectivity

Connections between the cerebellum and primary motor cortex (M1) can be evaluated with paired-pulse TMS. When a cerebellar conditioning stimulus precedes a test pulse over contralateral M1 by 5–7 ms, reductions in corticospinal excitability as indexed by a lower motor-evoked potential (MEP) amplitude can be observed in comparison to a single test pulse to M1 [84]. Daskalakis et al. [88] referred to this phenomenon as cerebellar inhibition (CBI). CBI involves the Purkinje cells of the cerebellar cortex and their inhibitory projections to the DCN. Signals from the DCN travel via the ventrolateral thalamus to cortical neurons in layers I, III, V, and VI [88]. TMS-induced activity in Purkinje cells causes suppression of excitatory DCN output and a net reduction in excitatory signals passing from the ventrolateral thalamic nucleus to M1 [89]. While CBI is a measure of functional connectivity in the cerebello-thalamo-cortical pathway, it is limited to M1, requiring other techniques, such as EEG, when studying functional anatomical relations between the cerebellum and “silent” cortical association areas. TMS-EEG studies quantify connectivity by time-locked traces of neural activity in the form of TMS-evoked neural activity. Alternatively, neural oscillations, which constitute the activity of synchronized fluctuations in membrane potentials of similarly oriented neurons that can be recorded by EEG [90], can be used to investigate signal transfer and connectivity.

Cerebellar TMS-EEG was likely first successfully implemented by Amassian, Cracco, Maccabee, and Cracco [83]. This study demonstrated that cerebellar TMS can elicit an evoked response in cortical brain regions as measured by EEG. Since then, different studies have employed a similar combination of techniques to explore the effects of cerebellar TMS on cerebral cortical activity. Notably, single-pulse TMS administered to the left and right posterolateral cerebellum has been used to investigate cerebellar connectivity with parietal and prefrontal regions [91–94]. The frequency of neural oscillations as a response to cerebellar stimulation can also be used to study cerebellar connectivity. Schutter and van Honk [94], for example, observed theta (4–7 Hz) EEG following a single-pulse TMS to medial cerebellum. This observation suggested the involvement of the cerebello-cortical pathway in emotional and cognitive processes [95, 96].

The effects of different TMS protocols on cerebellar connections have also been studied by evaluating functional connectivity between the cerebellum and M1. Repetitive TMS (rTMS), for example, applies short trains of multiple pulses to induce increased or reduced excitability of the cerebellum. The effects are dependent on the frequency of the stimulation, with low-frequency rTMS (≤ 1 Hz) generally eliciting inhibition while high-frequency rTMS (≥ 5 Hz) leads to excitation [97]. Oliveri et al. [98] demonstrated that excitability in M1 could be facilitated after 1 Hz rTMS of the contralateral cerebellum as evidenced by larger MEP amplitudes after stimulation. Other rTMS protocols like theta-burst stimulation (TBS) have also been shown to influence cerebellar functional connectivity. TBS applies high-frequency pulses over a short amount of time. More specifically, in continuous TBS (cTBS), triplets of 50 Hz pulses are applied every 0.2 s up to a total of 300 or 600 pulses, causing a decrease of cortical excitability in the targeted region. By contrast, for intermittent TBS (iTBS), 10 bursts of 50 Hz triplets are applied, followed by an 8 s interval of no stimulation, subsequently resulting in excitation of the targeted tissue [99, 100]. Koch et al. [101] and Popa et al. [102] showed that cerebellar cTBS reduced CBI and MEP amplitudes, while cerebellar iTBS increased MEP amplitudes, confirming the differential effects of TBS protocols on cerebellar connectivity. In short, these studies provide evidence that TBS can modulate cerebellar functional connectivity in a stimulation-dependent manner.

Lastly, cerebellar TMS has also been combined with other neuroimaging modalities to further elucidate cerebellar connectivity. Cho et al. [103], for instance, combined 1 Hz rTMS with positron emission tomography (PET) scans to show that metabolic changes occurred in non-motor areas like orbitofrontal, medial frontal, and anterior cingulate gyri, which are regions known to be involved in cognition and emotion.

In general, results of cerebellar TMS studies on functional connectivity are consistent with anatomical observations. Anatomical studies have found that cerebello-cerebral cortical communication occurs through closed loops of feedforward and feedback projections [104]. These pathways reciprocally connect the cerebellum to the primary motor and premotor areas of the cerebral cortex [105–107], as well as to non-motor cortical regions including the medial [108], the dorsolateral [106], and the anterior prefrontal cortex [109]. Similarly, cerebellar TMS studies have revealed connections between the cerebellum and motor and non-motor cortical areas.

#### Motor, Cognitive, and Affective Functions

In motor studies, effects of cerebellar TMS have been demonstrated in functions traditionally known to involve the cerebellum, such as coordination, monitoring, estimation, and correction of movements [110]. During the last decade, cerebellar TMS has also been shown to influence functions related to motor learning and motor adaptation. For motor adaptation, changes in CBI have been observed during visuomotor and locomotor adaptation tasks [111, 112]. Further, a recent study demonstrated that right cerebellar iTBS can improve visuomotor adaptation while cTBS disrupts adaptation [113]. In experimental motor learning paradigms, cTBS over the right cerebellum was reported to disrupt motor acquisition, retention, extinction, and re-acquisition of movements [114–116].

In cognitive studies, effects of stimulation have been primarily demonstrated in functions related to memory [117–119], time perception [120–124], and language [125–129]. It has also been noted that some of these cognitive processes are mostly lateralized in the cerebellum. Stimulation of the right cerebellum influences language processes, as well as functions related to time perception, whereas stimulation of the left cerebellum influences cognitive processes involved in visuospatial tasks [110].

Finally, evidence is accumulating that the cerebellum plays a role in affective processes [130]. Affective processes related to emotion regulation and attentional biases of emotional facial and body expressions can be modulated by cerebellar TMS. For example, disrupting cerebellar activity with low-frequency rTMS can lead to impairments in emotion regulation [131], whereas high-frequency rTMS is able to facilitate implicit processing of positive emotional stimuli in healthy volunteers [132]. More recently, Ferrari et al. [133] demonstrated that high-frequency rTMS over the left cerebellum impaired participants’ ability to discriminate between emotional faces. Consistent with these findings, left cerebellar TMS disrupted negative emotional content processing in a discrimination task for emotional body postures [134].

#### Clinical Applications

The therapeutic efficacy of cerebellar TMS to treat several motor dysfunctions has been investigated in neurological disorders [135]. Most notably, since Shimizu and colleagues [87] first demonstrated that rTMS alleviated gait ataxia in spinocerebellar ataxic (SCA) patients, various studies have explored the implementation of a wide variety of rTMS protocols to treat SCA [136]. Consequently, research during the last decades has found that modulation of cerebellar excitability by means of rTMS can elicit beneficial effects on several SCA domains [137–142]. Additionally, posterior cerebellar 1 Hz rTMS has been found to exert an anti-tremor effect on patients with essential tremors [143, 144]. In PD patients, cerebellar cTBS has been shown to improve levodopa-induced dyskinesia [145, 146], as well as positive effects of cerebellar cTBS on cervical dystonia [147–149].

The presence of cerebellar abnormalities in pathologies that affect cognitive and affective processes has sparked research into the potential of cerebellar TMS as a treatment for psychiatric populations. Most notably, cerebellar TMS has been shown to reduce negative and affective cognitive symptoms in schizophrenic patients [150–153]. In recent years, cerebellar rTMS has also been explored as a potential therapy for post-stroke rehabilitation. For example, cerebellar 1 Hz rTMS as well as iTBS have been shown to improve clinical scores of posture, gait, balance, and upper limb sensorimotor functionality as well as visuomotor learning in stroke patients [154–157]. Finally, cerebellar rTMS has been found to modulate human pharyngeal motor cortical excitability and can improve pharyngeal-related activity and swallowing behavior after transient disruption of the motor cortex [158]. Currently, clinical trials are underway to test the effects of cerebellar TMS for improving dysphagia in stroke patients [159].

### Cerebellar tDCS in the Twenty-First Century

While the use of transcranial polarization in research was gradually abandoned during the 1970s, interest in the method was rekindled 30 years later. At the turn of the century, two influential studies demonstrated that transcranially applied weak electric currents can influence the activation of the human motor cortex [160, 161]. This research led to a reappraisal of neural polarization, which is now more commonly known as transcranial direct current stimulation, as a tool for non-invasive neuromodulation [76]. Building on evidence from cerebellar TMS in the investigation of non-motor processes, the first study that applied cerebellar tDCS investigated the role of the cerebellum in a verbal working memory task [162]. Cerebellar tDCS during the last decade has been used to tackle the role of the cerebellum in motor, cognitive, and affective processes as well as in the treatment of neurological disorders. These studies have found similar results to cerebellar TMS studies.

#### Motor, cognitive, and affective functions

Cerebellar tDCS motor studies have explored the role of the cerebellum primarily in processes related to motor learning and motor adaptation. Anodal cerebellar tDCS has been reported to facilitate performance in various motor adaptation paradigms [163–168] with evidence suggesting that cathodal cerebellar tDCS may also inhibit adaptation [169]. Motor learning studies have found that anodal cerebellar tDCS can exert a facilitatory effect on the acquisition, retention/consolidation, and re-acquisition of motor sequences [170–174]. Some studies have also found that cathodal cerebellar tDCS can inhibit acquisition and retention [174, 175].

In the cognitive domain, effects of stimulation have been primarily demonstrated in functions related to working memory [162, 176–178] and language [179–183]. Although most of these studies have found cerebellar tDCS to influence these cognitive processes, the effects of tDCS on cognitive functions are much smaller than in motor functions [184]. Similarly, unlike motor studies that reported facilitatory effects of anodal and an inhibitory effects of cathodal cerebellar tDCS, polarity-dependent effects of cerebellar stimulation are far less obvious in cognitive studies [184].

Finally, tDCS is increasingly used in the investigation of the role of the cerebellum in affective processing. Evidence of cerebellar involvement in emotional processes related to the recognition of negative emotional faces has been reported by a study that found both cathodal and anodal cerebellar tDCS to facilitate this process [185]. Similarly, Newstead and colleagues [186] reported that frontocerebellar tDCS, with the anode placed over the left dorsolateral prefrontal cortex and the cathode over the right cerebellum, led to improved mood measures in healthy participants. Notably, the same study also observed that the opposite polarity montage elicited comparable effects on mood.

#### Clinical Applications

Similar to cerebellar TMS studies, cerebellar tDCS is primarily explored as a treatment for motor disorders. Mirroring results of cerebellar TMS studies, cerebellar tDCS was found to improve motor performance in SCA patients [187–192]. Further, preliminary results suggest that cerebellar tDCS can improve levodopa-induced dyskinesia [193] and balance [194] in PD patients. Lastly, cerebellar tDCS is increasingly being explored to enhance the recovery of stroke patients. These studies found that cerebellar tDCS can facilitate the recovery of some language functions [195, 196] and motor recovery in balance and gait performance [197–200] in post-stroke rehabilitation.

### Invasive Cerebellar Neurostimulation in the Twenty-First Century

While clinical attention shifted away from invasive to non-invasive cerebellar neurostimulation techniques at the turn of the last century, invasive forms of neuromodulation are still used in neurological patients [6]. For example, invasive cerebellar stimulation is being explored as a potential therapy for post-stroke rehabilitation. The extent of recovery in stroke survivors has been linked to many factors including cortical excitability during rehabilitation in the chronic phase of a stroke [201, 202]. In line with the existence of closed cerebello-cortical loops, cerebral cortical excitability can be enhanced by targeting the dentate nucleus of the DCN with chronic electric stimulation [203]. In stroke rodent models, chronic deep cerebellar stimulation has been shown to facilitate motor recovery [203] and promote neuroplastic reorganization [204]. Similar functional and motor improvements have been reported by optogenetic neuronal stimulation of the lateral cerebellar nucleus of mice [205]. Taken together, these studies suggest that deep cerebellar neurostimulation could potentially assist stroke patients in regaining lost functionality during rehabilitation [6].

In recent years, the cerebellum has also been considered a potential target in the treatment of dystonia. Sokal et al. [206], for instance, analyzed data of 10 patients suffering from cerebral palsy with spasticity who were treated with deep anterior cerebellum stimulation for 2 to 11 years. In this study, stimulation led to reduced symptoms of secondary dystonia in these patients. Consistent with this observation, White and Sillitoe [207] elicited dystonia-like symptoms in mice by genetically disruptin olivocerebellar excitatory neurotransmission, providing evidence for the central role of olivocerebellar communication in motor disorders. In the same study, the authors showed that pharmacological inhibition of cerebellar nuclei output helped to improve movement dysfunctions and that DBS of the interposed nuclei of the DCN instantaneously alleviated dystonia-like symptoms. Future research is needed to determine the viability of cerebellar DBS for dystonia and other motor dysfunctions in humans.

## Challenges and Future Directions

Cerebellar stimulation faces many of the same challenges that other emerging disciplines encounter during their initial years as well as unique challenges to the field. To further advance the field, future research may want to take into account a number of factors. First, the rapid expansion of the field has led to an exponential growth of the literature. Currently, a wide variety of experimental protocols and paradigms are used in noninvasive cerebellar neurostimulation research. In part, this can be understood by the fact that cerebellar stimulation as compared to cerebral cortical stimulation is still in its initial phase. In targeting the cerebellum, the majority of research relies on stimulation parameters, protocols, mechanisms, and safety that directly draws from cerebral cortical stimulation research. While this is understandable, applying this approach to the cerebellum which is architectonically and morphologically different from the cerebral cortex is not straightforward. In particular, when it comes to understanding the effects of electric and magnetic stimulation on the modulation of cerebellar functions, this limitation warrants future research about the basic neurophysiological mechanisms of cerebellar stimulation. The lack of detailed guidelines and standard practices in the field has likely contributed to variability of findings in these experiments. Further, a common complaint that has been echoed by different reviews on cerebellar TMS and tDCS [90, 110, 208] pertains to an obvious lack of standard protocols suitable for effective cerebellar stimulation. To increase the reliability of cerebellar stimulation studies, detailed guidelines and standard practices in terms of the protocols used for noninvasive cerebellar neurostimulation are necessary.

Secondly, there are several outstanding issues concerning the application of noninvasive cerebellar neurostimulation that may further contribute to the high degree of variability across individuals. The effects of invasive and non-invasive cerebellar stimulation depend on numerous aspects including stimulation parameters, differences in scalp-cortex distance, and cerebellar physiological variation in tissue susceptibility to electric currents and arousal level of the central nervous system. It has been shown that neurons within the same target area can be depolarized or hyperpolarized by the same electric field due to differences in neuronal orientation and shape of current flow [209]. Consequently, anatomical differences or inadequate electrode/coil placement may lead to heterogenous stimulation of the targeted tissue. Because of the high level of cortical folding in the cerebellum, relative small anatomical interindividual differences may have a greater influence on stimulation responses in the cerebellum compared to other brain regions [210]. Further, recent research has shown that individual factors like the amount of cerebrospinal fluid (CSF) and electrode-to-cortex distance also influence the neurophysiological outcomes of tDCS [211]. Currently, stimulation protocols are not optimal to account for these factors. Similar issues related to the depth of stimulation and focality of noninvasive brain stimulation techniques further limit the precision of stimulation procedures. A multimodal approach to cerebellar stimulation research might play an important role in complementing current protocols and/or in overcoming some of their limitations. Interleaving non-invasive brain stimulation with neuroimaging modalities can be used to study the temporal and spatial characteristics of neural activation in response to cerebellar neurostimulation [103, 179, 212, 213]. Consequently, a combined approach may provide new insights into how the cerebellum establishes its physiological and functional effects in the brain. Further, the precision of current protocols can also be improved by implementing a combined approach. For example, the use of neuronavigation may be able to better account for neuroanatomical differences across individuals as compared to fiducial landmarks on the head [102, 214–216]. Furthermore, computational modeling studies provide a powerful tool to examine individual electric field distributions, taking into account differences in MRI-derived anatomical features which may reduce the neurophysiological effect of the stimulation [217–221].

Thirdly, limitations of current non-invasive protocols are likely to remain an issue, yet alternative protocols and techniques of stimulation might yield new insights into aspects of cerebellar functioning for which current protocols are not optimized. For example, protocols capable to influence ongoing oscillatory cerebellar activity such as transcranial alternating current stimulation (tACS) [222] or cerebellar rTMS at particular frequencies may offer selective stimulation of neurons that oscillate in a frequency close to the stimulation frequency. Cerebellar oscillatory activity has been linked to several brain functions. For instance, facilitation of some motor functions has been reported after cerebellar tACS delivered at rhythmic patterns of > 30 Hz [223–225]. Similarly, cerebellar rTMS delivered to the posterior lobe in a theta rhythmic pattern (∼3–8 Hz) can influence cognitive functions related to memory whereas stimulation in beta rhythmic pattern (∼13–30 Hz) can influence some language-related processes [128]. Consequently, these techniques could potentially offer an alternative to modulate cerebellar activity [222]. Systematic studies of rhythm-specific effects of neurostimulation may enhance our understanding of cerebellar-related processes and functions. Future research will undoubtedly provide further insights into the experimental and clinical range of these methods.

Fourthly, while current stimulation protocols are considered to be safe and tolerable, future research will need to further address safety and tolerability of cerebellar-specific protocols across a wider range of stimulation parameters. The majority of protocols currently used in the field are direct adaptations from protocols used in the cerebral cortex, which might not be optimal for cerebellar stimulation. Because there is an interdependent relationship between stimulation parameters and stimulation depth and focality [208], future research aiming at developing cerebellar-specific protocols might explore the feasibility and efficacy of using more intense parameters of stimulation. While preliminary studies are already exploring the effects of more intense stimulation parameters for cerebellar tDCS [194] and TBS [226], safety and tolerability aspects of these procedures are not always documented. This is far from a recent problem, as previous reviews have remarked that adverse events following stimulation are inconsistently reported by both invasive and non-invasive cerebellar studies [208, 227]. Consequently, there is some concern that developing cerebellar-specific protocols might lead to reduced tolerability and higher discomfort of stimulation procedures. In order to safely advance current protocols for cerebellar neurostimulation, future studies may want to adopt a consistent practice of reporting on the safety and presence of adverse events following stimulation.

A final issue is the growing interest of invasive and non-invasive cerebellar stimulation to treat different motor and psychiatric disorders. Currently, there is no consensus on which cerebellar areas should be targeted for different diseases and when the cerebellum as a target site should be preferred over other brain areas [6]. Further, low replicability of cerebellar neurostimulation studies might hinder their validity within a clinical context where robust effects are especially desirable [228]. Nevertheless, future clinical and experimental research will likely further elucidate the role of the cerebellum in existing psychopathological models. Similarly, adequately powered randomized controlled trials may offer further insights into the clinical usefulness of cerebellar neurostimulation.

## Conclusion

The present review has provided a brief historical overview of the development of cerebellar neurostimulation techniques. Modern techniques of cerebellar stimulation have helped to elucidate cerebellar contributions to diverse motor, cognitive, and affective processes. Presently, the field still faces several challenges, but advancing multidisciplinary efforts, combining techniques, and standardizing protocols for adequate cerebellar stimulation may contribute to addressing some of these issues. Additionally, exploring new methods and protocols for cerebellar stimulation might advance our understanding of cerebellar functions and anatomy by exploiting alternative methods of stimulation. However, future research exploring new protocols may benefit from a standardized practice on safety-related issues. Finally, much remains unknown on the role of the cerebellum in different psychopathologies and further clinical research is warranted to establish the potential of cerebellar neurostimulation in clinical practice.
